# Coupling Effects of Environmental Factors on the Phytoplankton Community Structure in Ebinur Lake, a Saltwater Lake in China

**DOI:** 10.1002/ece3.71516

**Published:** 2025-06-14

**Authors:** Huibo Wang, Le Wang, Xue Du, Xiaoli Huang, Chen Zhao, Zhongsi Gao, Tangbin Huo

**Affiliations:** ^1^ Heilongjiang River Fishery Research Institute Chinese Academy of Fishery Sciences Harbin China; ^2^ Key Laboratory of Aquatic Organism Protection and Ecological Restoration in Cold Waters Harbin China; ^3^ Sichuan Agricultural University Chengdu China

**Keywords:** coupling effect, environmental factor, high salinity lake, phytoplankton community structure

## Abstract

Studying the coupled effects of environmental factors on the structure of phytoplankton communities can deepen our understanding of the stability of aquatic ecosystems in extreme environments. This study examined the phytoplankton community structure and environmental factors of saline a lake during spring, summer, and autumn in 2019. A total of 95 phytoplankton species (belonging to 47 genera and 7 phyla) were identified in Ebinur Lake, reflecting a species richness lower than those of freshwater lakes while being greater than the levels observed in other saltwater lakes. Bacillariophyta dominated the phytoplankton assemblage, followed by Chlorophyta and Cyanophyta, with lesser diversity in other algal species, suggesting that the species composition was similar to that observed in other saltwater lakes. There was considerable spatiotemporal variation in the structure of the phytoplankton community, with the biomass of phytoplankton displaying notable seasonal variation. In spring, the biomass of Bacillariophyta was dominant; in summer, as the climate warmed, the biomass of phytoplankton reached its peak and the biomass of Chlorophyta was dominant; in autumn, the biomass was the lowest, and Chlorophyta and Bacillariophyta shared dominance. The spatial distribution was relatively consistent, as reflected in the distribution of phytoplankton in the three seasons, with the southeastern area of the lake generally exhibiting higher biomass than other lake areas. Bacillariophyta and Chlorophyta were significantly correlated with water transparency (SD); Cyanophyta was significantly correlated with water temperature (WT), and Cryptophyta was significantly correlated with pH. The interaction effects of various environmental factors, including pH, SD, Chlorophyll‐a (Chl‐a), ammonia nitrogen (NH_4_^+^‐N), and salinity (S), jointly affect the dynamics of the phytoplankton community structure in Ebinur Lake. This study investigated the effects of physicochemical factors on the structure of the phytoplankton community in a high salinity lake, thereby providing a basis for ecological protection and environmental management of aquatic ecosystems in extreme environments.

## Graphical abstract

This study examined the phytoplankton community structure and environmental factors of a saltwater lake in China, and investigated the effects of physicochemical factors on the structure of the phytoplankton community. The interaction effects of various environmental factors, including pH, SD, Chl‐a, NH_4_^+^‐N, and salinity, jointly affect the dynamics of the phytoplankton community structure of the lake.

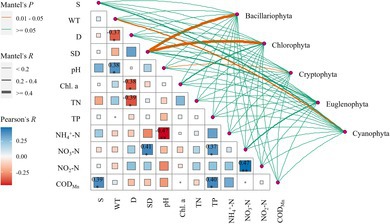

## 1 Introduction

As the primary producers in aquatic ecosystems, phytoplankton are highly sensitive to environmental changes and are intimately linked to the nutritional status of water bodies. Phytoplankton are involved in the ecological processes of material transformation, energy flow, and information transfer in water bodies (Dokulil and Qian 2021; Falkowski 2012; Field et al. 1998; Mehner et al. 2008). As essential forage organisms, the abundance of phytoplankton has important implications for lake fishery production. Phytoplankton form the foundation of lake food webs, with their productivity supporting higher trophic levels such as zooplankton and fish, thereby influencing the energy flow and material cycling within the entire ecosystem (Hamer et al. 2022; Titocci et al. 2022).

The composition and structure of phytoplankton communities are influenced by a myriad of environmental factors, including temperature, pH, salinity, and nutrient concentrations of water bodies (Larson and Belovsky 2013; Sugie et al. 2020). In addition to independently affecting the growth and distribution of phytoplankton, these factors have coupling effects on the community structure via complex interactions. The exploration of these coupling effects is important not only for predicting the dynamic changes in phytoplankton communities but also for gaining insight into the stability of aquatic ecosystems in general. Morphological characteristics of phytoplankton, such as cell shape, surface‐to‐volume ratio, and maximum axial linear dimension, exhibit specific distribution patterns across different biogeographical regions and water column depths. These traits are closely linked to the availability of light and nutrients, enabling phytoplankton to adapt to diverse ecological conditions (Paul and Patil 2024). Environmental changes, including rising temperatures, nutrient fluctuations, and alterations in hydrological conditions, can lead to shifts in phytoplankton community structure, affecting their biomass, species composition, and diversity (Chen et al. 2023; Tian et al. 2017; Verbeek et al. 2018; Yang, Chen, et al. 2022). Some studies have also indicated that the sensitivity of phytoplankton to environmental changes varies with latitude (Chaffron et al. 2021), light drives phytoplankton productivity, so phytoplankton must exploit variable intensities and durations of light exposure, depending upon season, latitude, and depth (Li et al. 2016). Additionally, a high salinity was also an adverse factor for phytoplankton (Jia et al. 2022). Different phytoplankton taxa exhibit varying tolerances to salinity; thus, analyzing community dynamics can reveal the patterns of salinity evolution in lakes (Olofsson et al. 2020). As an important environmental parameter of plateau lake systems, salinity directly affects physiological phytoplankton processes. An increase in salinity can affect the osmotic pressure of phytoplanktonic cells, causing the dysfunction in ion absorption processes of these cells in aquatic environments (Li, Gao, et al. 2021; Redden and Rukminasari 2008).

This study focuses on Ebinur Lake, a saline lake located on the Chinese plateau, characterized by both high salinity and seasonal ice coverage. Investigating the planktonic community structure in highland saline lakes holds particular ecological significance and offers a theoretical foundation for understanding how aquatic organisms adapt to extreme environments. Ebinur Lake, the largest saltwater lake in Xinjiang, China, spans 650 km^2^, with an average depth of 1–2 m and a surface elevation of 189 m. As an ecological barrier on the western edge of the Junggar Basin, Ebinur Lake and its associated wetlands play a critical role in mitigating desertification, controlling the spread of sandstorms, and maintaining the ecological stability of surrounding oases. Moreover, Ebinur Lake is one of Xinjiang's key production areas for *Artemia salina* , which feed on phytoplankton. Therefore, research on phytoplankton not only contributes to understanding the ecosystem but also underpins the sustainable exploitation of *Artemia salina* resources. As primary producers, phytoplankton support zooplankton such as *Artemia salina* and filter‐feeding fish, which in turn sustain avian species, including migratory waterbirds, thereby structuring the entire desert wetland food web. In recent years, intensified climate change and human activities have exacerbated aridification in Xinjiang, leading to a reduction in lake area, increased salinity, rapid lake surface shrinkage, and worsening water pollution. Changes in phytoplankton community structure, such as increased dominance of salt‐tolerant species, can serve as direct indicators of regional climate change. Furthermore, the microbial resources in the lake require urgent investigation and conservation. From an economic development perspective, Ebinur Lake holds significant potential due to its high salinity, which supports rich populations of *Dunaliella salina* , one of the richest natural sources of β‐carotene (accounting for over 10% of its dry weight). In addition, Ebinur Lake has an annual *Artemia salina* resource yield of approximately 4000 t, ranking highest among Chinese saltwater lakes. *Artemia salina* are rich in protein and essential amino acids, making them a high‐quality feed for aquatic animal larvae. Both *Dunaliella salina* and *Artemia salina* are valuable biological resources that can support the development of an integrated “algae‐ *Artemia salina* ‐deep processing” industrial chain (Bai 2010). However, alongside resource exploitation, equal emphasis must be placed on the conservation and management of the lake's biological and environmental integrity.

The environmental factors present in water bodies and their interactions have multifaceted and complex impacts on the structure of phytoplankton communities. The changes in environmental factors not only affect the species composition and abundance of phytoplankton but also play a pivotal role in the health status of aquatic ecosystems. Although numerous studies have explored the relationships between lake phytoplankton and environmental factors, there is a notable lack of research addressing phytoplankton responses to extreme environmental conditions in saline lakes that experience seasonal ice cover. Furthermore, there has been a notable absence of research on the community structure of planktonic organisms in Ebinur Lake. Through studying the coupling effects of environmental factors on the structure of phytoplankton communities in Ebinur Lake, the present study aims to elucidate the structure, species composition, and spatiotemporal distribution of phytoplankton communities in the lake and their correlations with environmental factors, and to infer the mechanism by which physicochemical factors drive the seasonal variation in dominant phytoplankton taxa. Conducting this research is not only critical for understanding the ecological dynamics of inland saline lakes in China, but also offers scientific support for water quality assessment, climate change adaptation, and biological resource management. Additionally, it provides theoretical guidance for ecological interventions such as lake ecological water replenishment and saline‐alkali land remediation. The lake's distinctive high‐salinity conditions also make it a valuable case study for investigating planktonic life in extreme environments.

## 2 Materials and Methods

### 2.1 Study Area

Located in the northwestern part of Xinjiang, China (43°38′–45°52′N, 79°53′–85°02′E), Ebinur Lake is encircled by mountains from the west, north, and south, and its east side adjoins the Junggar Basin at an altitude of 154 m to 4827 m and a drainage area of 50,621 km^2^. The climate in this region is classified as temperate continental arid, characterized by abundant sunshine and extreme temperature changes, with an annual evaporation of approximately 2221.3 mm and an annual precipitation of about 105.17 mm. The maximum temperature is 42.23°C; the minimum temperature is −36.4°C, and the annual sunshine duration is 2699.87 h (He et al. 2017; Yushanjiang et al. 2018). Ebinur Lake is considered Xinjiang's largest saltwater lake, with a surface area of 542 km^2^. The main sources of water are separately the Bortala River flowing from the west and the Jinghe River flowing from the south.

As a crucial ecological barrier not only for Xinjiang but also for the entire northwest region of China, Ebinur Lake provides numerous invaluable contributions. The health of its water environment is associated not only with the local economy but also with the sustainable development of the basin. Unfortunately, recent years have seen the lake encountering non‐point source pollution that has damaged the environment of its basin due to agricultural production, urbanization, and human intervention (Amiri and Nakane 2009; Xiao et al. 2016).

### 2.2 Sampling Time and Sample Point Layout

**FIGURE 1:**
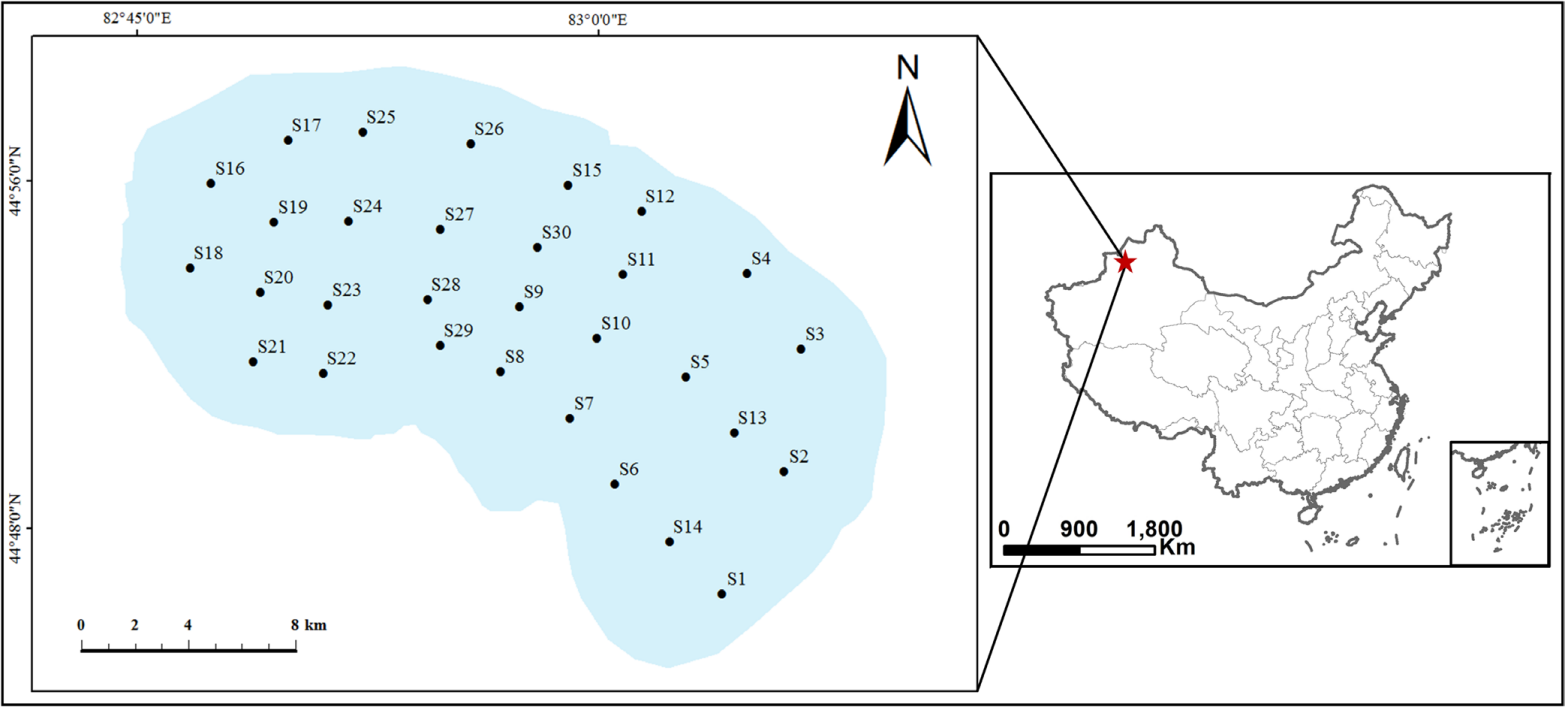
Locations of sampling sites of Ebinur Lake.

Given that Ebinur Lake, a shallow lake at a relatively high latitude, experiences a 4‐month ice‐bound period during winter, the phytoplankton community structure and environmental factors of the lake during spring (May), summer (July), and autumn (October) in 2019 were explored, with no water samples collected during the winter months. Finally, 30 sampling points were established across the lake (Figure 1).

### 2.3 Sample Collection and Processing

At each sampling site, one qualitative and one quantitative phytoplankton sample were collected, yielding a total of 60 samples. Qualitative samples were collected using a 5 L organic glass water collector. From each site, 1 L of water was obtained. Each sample was fixed and stained with Lugol's iodine solution and then brought back to the laboratory. After 48 h of sedimentation via the precipitation method, the supernatant was removed, and a 30 mL concentrated solution was used to identify the phytoplankton. Qualitative samples were collected by plankton net (64 μm mesh size), and samples were fixed on‐site with 4% formaldehyde.

For quantitative analysis, the sample was mixed thoroughly, and 0.1 mL of the water sample was quickly extracted using a pipette and placed into a 0.1 mL counting chamber (with an area of 20 mm ×20 mm). A cover slip was placed on top, and phytoplankton were identified and counted under a compound microscope at 400× magnification (Echo, RVL‐100, USA). Two slides were prepared for each sample, and the final count was calculated as the average of the two. Qualitative and quantitative analyses were conducted on the phytoplankton according to “Research Methods for Freshwater Phytoplankton” (Zhang and Huang 1991) and “Aquatic Biology” (Zhao 2005). In addition, the identification of phytoplankton species was conducted based on “Chinese Freshwater Algae – Systems, Classification, and Ecology” (Hu and Yinxin 2006).

On‐site measurements of the water temperature (WT), pH, and salinity (S) were made using a YSI multi‐parameter water quality analyzer model EXO2. The water transparency (indicated by secchi depth (SD)) was measured using a Secchi disk, and a Speedtech portable depth sounder was employed to measure the water depth (D). The chlorophyll‐a (Chl‐a) content was measured by the 90% acetone extraction method. Water samples collected on‐site were used to measure total nitrogen (TN), total phosphorus (TP), ammonia nitrogen (NH_4_^+^‐N), nitrate nitrogen (NO_3_–N), nitrite nitrogen (NO_2_–N), and permanganate index (COD_Mn_) (Ministry of Environmental Protection (MEP) China 2002).

### 2.4 Data Processing

#### 2.4.1 Dominance

Dominance was used to evaluate the main species of phytoplankton. The calculation equation is:

(1) $$Y=f_{i}\times P_{i}$$

where *Y* is the dominance; *f*_i_ is the frequency of occurrence of the *i*‐th species, and *P*_*i*_ is the proportion of the number of individuals of the *i*‐th species in the total number of individuals. The species whose *Y* values were > 0.02 were classified as dominant species (Aksnes and Wassmann 1993).

#### 2.4.2 Comprehensive Trophic Level Index (TLI) Method

TLI is a comprehensive eutrophication evaluation method taking Chl‐a, TP, TN, SD, and COD_Mn_ as the evaluation indicators. The TLI model is as follows (Wang et al. 2019):

(2) $$\begin{array}{c} \mathrm{TLI}\left(\sum \right)=\sum_{j=1}^{m}W_{j}\times \mathrm{TLI}\left(j\right) \end{array}$$

where TLI (Ʃ) is the comprehensive trophic level index, TLI (*j*) represents the trophic state index of parameter *j*, *W*_*j*_ represents the corresponding weight of parameter *j*, and *m* represents the number of evaluation parameters.

Take Chl‐a as the benchmark parameter, the normalized correlation weight of parameter *j* is as follows:

(3) $$\begin{array}{c} W_{j}=\frac{r_{\mathrm{ij}}^{2}}{\sum_{j=1}^{m}r_{\mathrm{ij}}^{2}} \end{array}$$

where *W*_*j*_ represents the corresponding weight of parameter *j*. *r*_*ij*_ represents the correlation coefficient between benchmark parameter (Chl‐a) and parameter *j*. *m* is the number of evaluation parameters.

Trophic level indexes of each parameter are calculated as Equations ((4), (5), (6), (7), (8)).

(4) $$\begin{array}{c} \mathrm{TLI}\left(\mathrm{Chl}-a\right)=10\times \left(2.5+1.086\times \ln\;\mathrm{Chl}-a\right) \end{array}$$

(5) $$\begin{array}{c} \mathrm{TLI}\left(\mathrm{TP}\right)=10\times \left(9.436+1.624\times \ln\;\mathrm{TP}\right) \end{array}$$

(6) $$\begin{array}{c} \mathrm{TLI}\left(\mathrm{TN}\right)=10\times \left(5.453+1.694\times \ln\;\mathrm{TN}\right) \end{array}$$

(7) $$\begin{array}{c} \mathrm{TLI}\left(\mathrm{SD}\right)=10\times \left(5.118-1.94\times \ln\;\mathrm{SD}\right) \end{array}$$

(8) $$\begin{array}{c} \mathrm{TLI}\left({\mathrm{COD}}_{\mathrm{Mn}}\right)=10\times \left(0.109+2.661\times \ln\;{\mathrm{COD}}_{\mathrm{Mn}}\right) \end{array}$$

Based on a water eutrophication survey of 26 Chinese lakes, the reference value of correlation coefficients *r*_*ij*_ is as follows (Jin et al. 1995): *r*_Chl‐a_ = 1, *r*_TN_ = 0.82, *r*_TP_ = 0.84, *r*_SD_ = 0.83, *r*_CODMn_ = 0.83. Units are as follows: Chl‐a (μg/L), TP (mg/L), TN (mg/L), CODMn (mg/L), and SD (m).

The trophic state of the lakes is assigned as follows: TLI (Ʃ) scores of < 30, oligotrophic; TLI (Ʃ) scores of 30 to 50, mesotrophic; TLI (Ʃ) scores of 50 to 60, light eutrophic; TLI (Ʃ) scores of 60 to 70, medium eutrophic; TLI (Ʃ) scores of > 70, severe eutrophic.

#### 2.4.3 Statistical Analysis

R software was adopted to generate a species composition classification diagram for the phytoplankton in Ebinur Lake. Mantel tests of environmental factors and phytoplankton divisions were used to analyze the correlations between environmental factors in Ebinur Lake and the relationships between environmental factors and dominant divisions.

Redundancy Analysis (RDA) was conducted using the CANOCO (version 5.0) software to investigate the correlations between phytoplankton species and environmental factors (Šmilauer and Lepš 2014). The RDA analysis was conducted on phytoplankton species that appeared with a frequency above 13%. For the RDA analysis, environmental factor data were log (*x* + 1) transformed except for pH (Flores and Barone 1998).

ArcGIS was used to illustrate general trends of phytoplankton biomass by using Inverse Distance Weighting (IDW) (Liu and Wang 2001; Pflaumann et al. 1996).

## 3 Results

### 3.1 Structural and Spatiotemporal Distribution Characteristics of the Phytoplankton Community in Ebinur Lake

#### 3.1.1 Species Composition of Phytoplankton in Ebinur Lake

**FIGURE 2:**
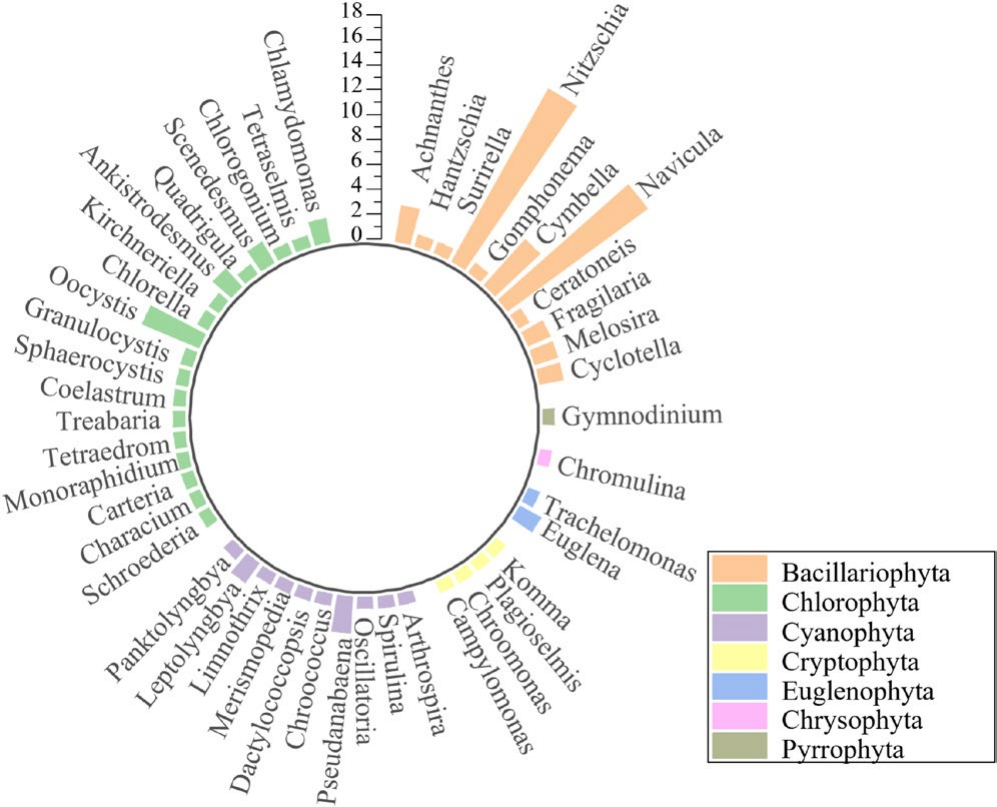
Species composition of the phytoplankton of Ebinur Lake.

A total of 95 phytoplankton species belonging to 47 genera and seven phyla were identified in Ebinur Lake. The phylum Bacillariophyta had the highest number of species (48), accounting for 50.5% of the total. The Chlorophyta division had 25 species and accounted for 26.3%, and the division Cyanophyta had 13 species and accounted for 13.7%. There were fewer species in the divisions Cryptophyta, Euglenophyta, Pyrrophyta, and Chrysophyta, accounting for 4.2%, 3.2%, 1.1%, and 1.1%, respectively (Figure 2).

A total of 25 phytoplankton species belonging to 17 genera and 5 phyla were found in spring, and the dominant species were *Oocystis* sp., *Navicula viridula* , and *Nitzschia stagnorum* . Accordingly, 54 species of phytoplankton belonging to 35 genera and 7 phyla were identified in summer, and the dominant species was *Dunaliella salina* . In addition, 42 species of phytoplankton belonging to 24 genera and 5 phyla were identified in autumn, and the dominant species were *Navicula viridula* and *Dunaliella salina* .

#### 3.1.2 Spatiotemporal Distribution of Phytoplankton Biomass in Ebinur Lake

**FIGURE 3:**
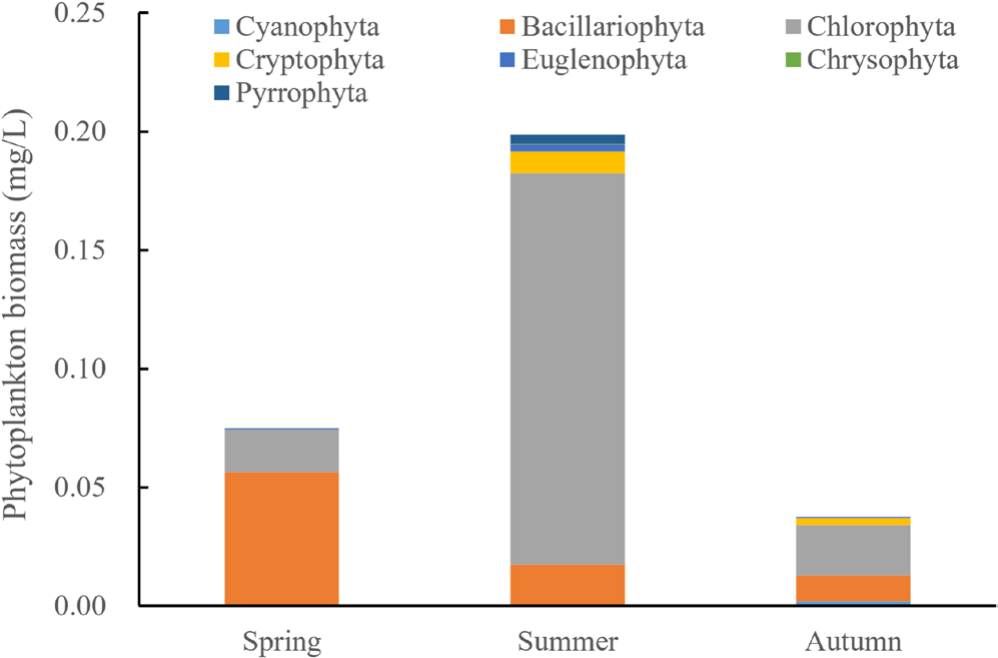
Seasonal variation in the phytoplankton biomass in Ebinur Lake.

The annual average biomass of phytoplankton in Ebinur Lake was 0.1035 mg/L. There is significant seasonal variation in the phytoplankton biomass in Ebinur Lake. During spring, the biomass was 0.0747 mg/L, with the Bacillariophyta dominating the distribution at 0.0564 mg/L. As the summer progresses and the climate warms, the phytoplankton biomass surges to its peak of 0.1986 mg/L, primarily driven by the proliferation of the Chlorophyta at 0.1649 mg/L. In autumn, there was a decline in the phytoplankton biomass to its lowest point of 0.0372 mg/L, where Chlorophyta (0.0212 mg/L) and Bacillariophyta (0.0109 mg/L) shared a co‐dominant status (Figure 3).

**FIGURE 4:**
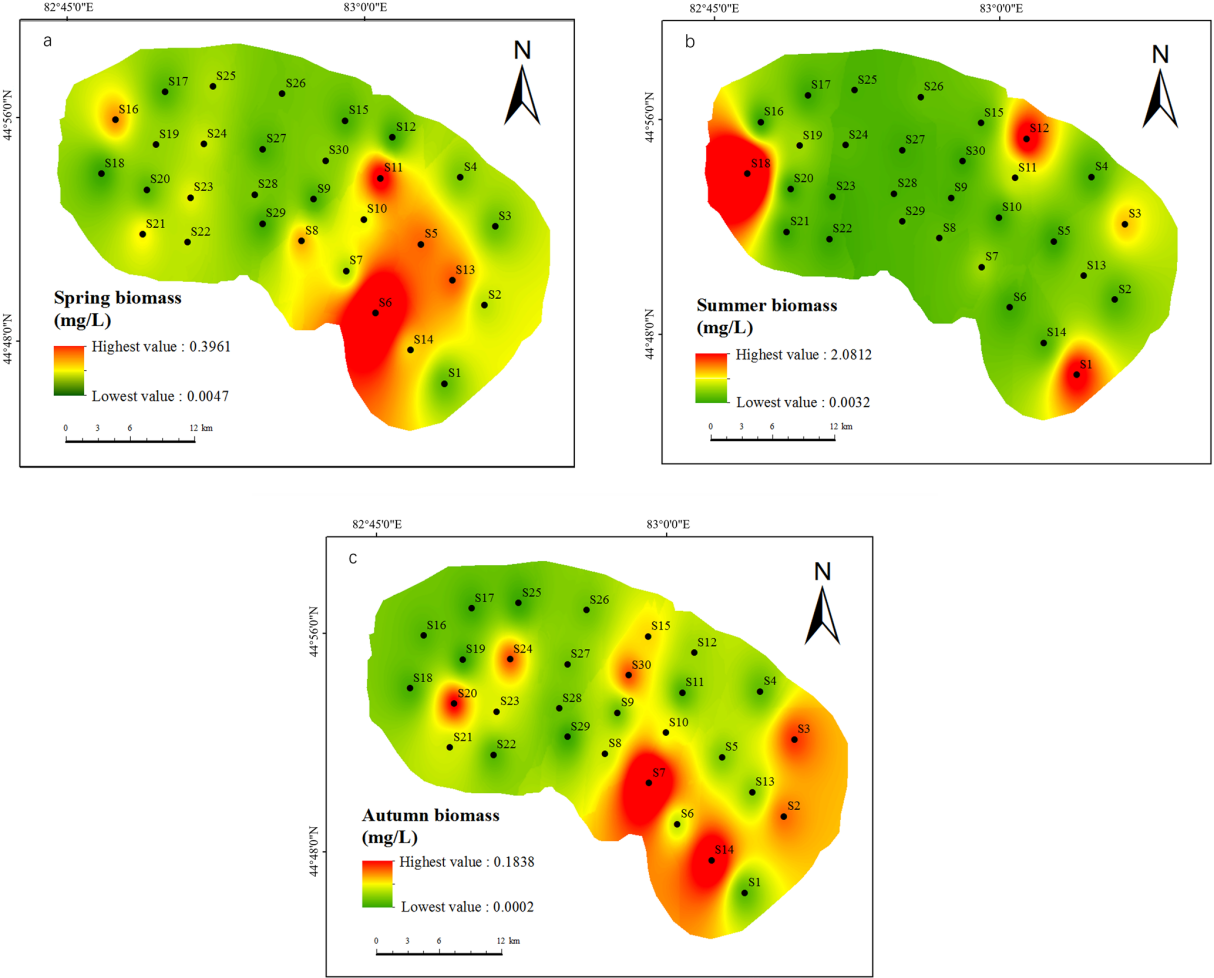
Interpolation results by Inverse Distance Weighting (IDW) of the phytoplankton biomass in (a) spring, (b) summer, (c) autumn in Ebinur Lake.

The spatial distribution of phytoplankton remained relatively consistent across the three seasons, with the sampling points in the southeast of the lake having a higher proportion of phytoplankton than other areas of the lake. The southeastern part of the lake is bordered by farmland and salt pans, so the characteristic spatial distribution of phytoplankton may be associated with the non‐point source pollution of the lake (Figure 4). During summer, the sampling sites S18, S12, and S1 consistently displayed remarkably high phytoplankton biomass, mirroring their elevated chlorophyll concentrations. *Dunaliella salina* emerged as the predominant species at the three sampling sites.

### 3.2 Spatiotemporal Variation of Environmental Factors in Ebinur Lake

Table 1 lists the environmental factors measured in Ebinur Lake (Table 1). The significance of these factors was explored using one‐way ANOVA. The results indicated significant seasonal variation in all factors except for NO_3_‐N (*p* > 0.05). During the summer at Ebinur Lake, WT, S, Chl‐a, NO_2_–N, and COD_Mn_ were significantly higher than in spring and autumn, while SD, pH, and NH_4_^+^‐N were relatively lower in summer compared to other seasons. In addition, autumn saw comparatively higher TP and TN. TLI indicated that the trophic states of Ebinur Lake were medium eutrophic. The TLI in summer was significantly higher than that in spring and autumn.

**TABLE 1:** Seasonal variation in environmental factors in the Tiehala Sub‐lake of Ebinur Lake.

Environmental factor	Spring	Summer	Autumn	*p*
S (‰)	10.05 ± 0.48	14.55 ± 3.55	12.65 ± 1.51	0.000
WT (°C)	19.68 ± 0.72	27.62 ± 1.37	11.87 ± 0.45	0.000
D (m)	1.52 ± 0.5	1.13 ± 0.43	1.03 ± 0.27	0.000
SD (m)	0.31 ± 0.12	0.16 ± 0.07	0.33 ± 0.07	0.000
pH	8.22 ± 0.05	8.08 ± 0.26	8.13 ± 0.08	0.004
TN (mg/L)	3.27 ± 0.71	7.03 ± 0.46	7.64 ± 3.14	0.000
TP (mg/L)	0.13 ± 0.04	0.17 ± 0.03	0.19 ± 0.03	0.000
NH_4_^+^‐N (mg/L)	0.84 ± 0.4	0.25 ± 0.05	0.89 ± 0.12	0.000
NO_3_–N (mg/L)	2.28 ± 0.3	2.17 ± 0.28	2.12 ± 0.16	0.065
NO_2_–N (mg/L)	0.12 ± 0.01	0.54 ± 0.06	0.29 ± 0.01	0.000
COD_Mn_ (mg/L)	6.98 ± 1.26	12.56 ± 3.90	10.56 ± 3.52	0.000
Chl‐a (μg/L)	9.13 ± 8.15	20.21 ± 20.59	8.03 ± 3.97	0.001
TLI	61	72	66	
Trophic state	Medium eutrophic	Severe eutrophic	Medium eutrophic	

### 3.3 Coupling Effects of Environmental Factors on the Structure of Phytoplankton Communities

**FIGURE 5:**
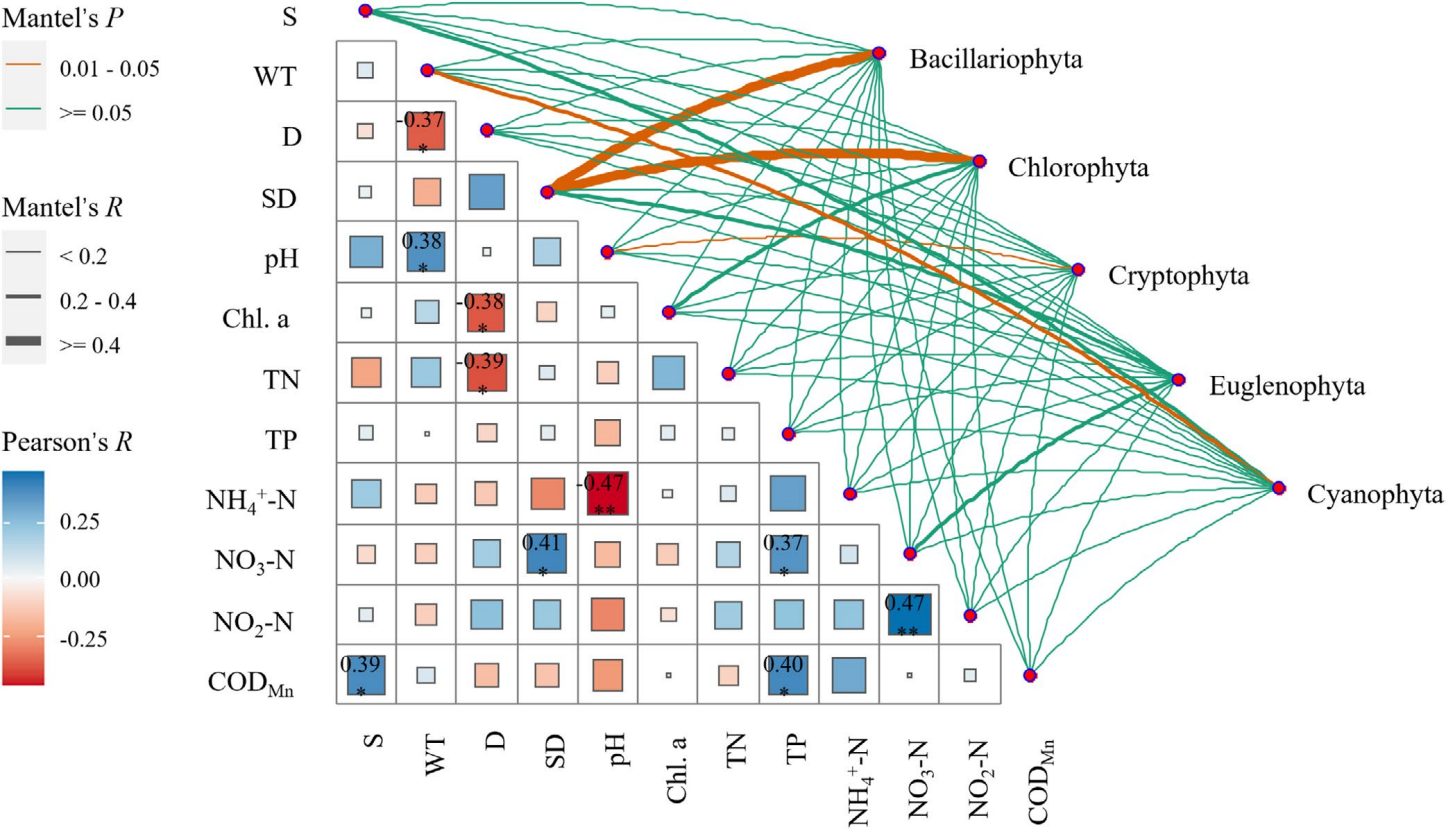
Mantel tests of environmental factors and phytoplankton phyla.

The correlations between environmental factors and their relationships with various categories of phytoplankton were investigated using Mantel tests and correlation heatmaps (Figure 5). Among the environmental factors, NO_3_–N was positively correlated with SD (*R* = 0.41, *p* < 0.05) and NO_2_–N (*R* = 0.47, *p* < 0.05); pH was negatively correlated with NH_4_^+^‐N (*R* = −0.47, *p* < 0.05); COD_Mn_ was positively correlated with TP (*R* = 0.40, *p* < 0.05) and S (*R* = 0.39, *p* < 0.05), and D exhibited significant negative correlations with TN (*R* = −0.39, *p* < 0.05) and Chl‐a (*R* = −0.38, *p* < 0.05). Bacillariophyta and Chlorophyta were significantly correlated with SD; Cyanophyta was significantly correlated with WT, and Cryptophyta was significantly correlated with pH.

**FIGURE 6:**
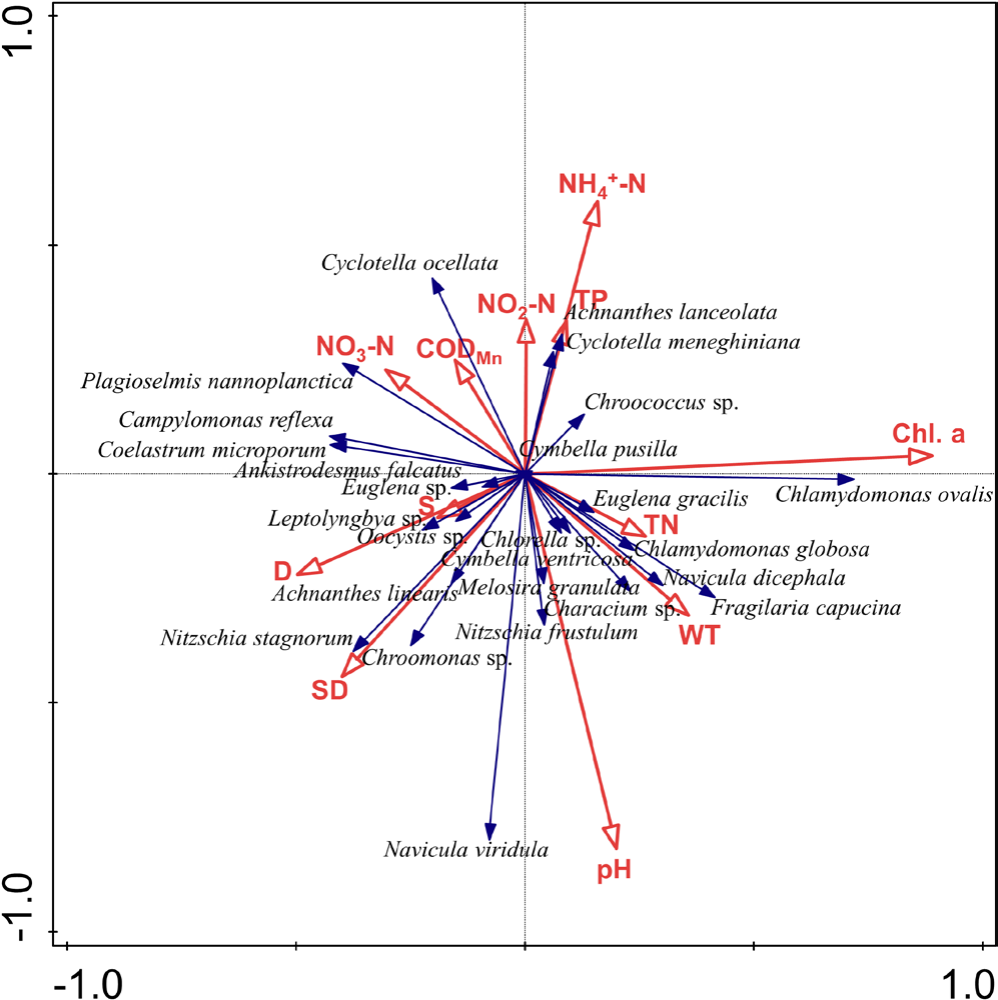
Ordination biplot from a Redundancy Analysis (RDA) between phytoplankton species and lake water environmental factors.

We used RDA to explore the relationship between species of phytoplankton and environmental factors (Figure 6). Axes 1 and 2 explained 13.17% and 23.37% of the variation in the zooplankton dominant species assemblages, respectively (*p* = 0.002, Monte‐Carlo permutation test using 999 permutations).

The RDA showed that Chl‐a, pH, SD, and NH_4_^+^‐N had significant correlations with the dominant species of the lake. The first axis showed a strong positive correlation with Chl‐a. The second axis showed a strong positive correlation with NH_4_^+^‐N and a strong negative correlation with pH. *Dunaliella salina* was strongly positively correlated with Chl‐a, and *Navicula viridula* was highly correlated with pH. *Nitzschia stagnorum* and *Chroomonas* sp. were strongly positively correlated with SD.

## 4 Discussion

### 4.1 Structure and Spatiotemporal Distribution Characteristics of the Phytoplankton Community in Ebinur Lake

The S of Ebinur Lake averages 12.42‰ annually, which classifies it as a slightly saltwater lake. In our investigation, 95 species of phytoplankton were recorded, reflecting a species richness lower than that of freshwater lakes while exceeding the levels observed in numerous other saltwater lakes (Chen et al. 2013; Huo et al. 2005; Shi et al. 2023; Yang, Gao, et al. 2022). Specifically, Bacillariophyta dominated the phytoplankton assemblage in Ebinur Lake, followed by Chlorophyta and Cyanophyta, with lesser diversity in other algal species, suggesting that the species composition was very similar to other saltwater lakes, for example, in the lakes of China's Qinghai‐Tibet Plateau (43 species of phytoplankton were identified, with Bacillariophyta and Chlorophyta dominating), Russia's Transbaikal Lake (73 species were found, dominated by Cyanophyta and Bacillariophyta), and five saltwater lakes in the Tibet region (53 species were recorded, dominated by Bacillariophyta, Chlorophyta, and Cyanophyta) (Afonina et al. 2025; Li, Gao, et al. 2021; Shi et al. 2023). Bacillariophyta, Chlorophyta, and Cyanophyta exhibit robust environmental resilience, enabling them to thrive as dominant populations across diverse water environments, including those with high S levels and challenging climatic conditions (Popescu and Ibnescu 2014). Therefore, these three algal divisions predominate in saline water bodies, while varying in their proportional representation across saline ecosystems. The dominant phytoplankton species in Ebinur Lake belonged to the Bacillariophyta and Chlorophyta, with Bacillariophyta including *Navicula viridula* and *Nitzschia stagnorum* and Chlorophyta such as *Dunaliella salina* and *Oocystis* sp. In a study investigating phytoplankton responses to nutrient levels along salinity and elevation gradients on the Qinghai‐Tibet Plateau, Li, Gao, et al. (2021) found a significant negative correlation between phytoplankton abundance and salinity (*p* < 0.05). Furthermore, Bacillariophyta and Chlorophyta exhibited higher salinity tolerance thresholds, which supports their survival in high‐salinity lake environments. During summer, as the WT rises, the lake is more suitable for phytoplankton growth, with the greatest number of species occurring in the survey. However, *Dunaliella salina* , the species with the highest degree of dominance, experiences rapid proliferation during summer, and Chlorophyta have an advantage. *Dunaliella salina* , a typical saltwater species, has high S tolerance and represents a key species in many saltwater lakes across China (Huo et al. 2005; Shi et al. 2023). Chlorophyta also have temperature tolerance, facilitating their proliferation during warmer months to become the dominant species. In contrast, Bacillariophyta are generally cold‐adapted and can grow in a cooler environment (Morais et al. 2003). Therefore, during spring and autumn when the WT is lower and temperature differences are more pronounced, the dominance of Bacillariophyta and Chlorophyta remains relatively stable.

Our investigation of the phytoplankton species in Ebinur Lake identified only one typical saltwater species, *Dunaliella salina* , a species that rarely occurs in freshwater. This is due to its high S tolerance; high S contributes not only to its growth but also to the production of high levels of β‐carotene (Oren 2014). The majority of phytoplankton detected in Ebinur Lake belonged to the euryhaline species or salt‐loving species such as *Chlorella* sp., *Oscillatoria tenuis* , *Kirchneriella obesa* in the Bacillariophyta, Chlorophyta, and Cyanophyta, species that are well adapted to S, echoing the findings of other saltwater bodies (Servant‐Vildary and Roux 1990; Wood and Talling 1988; Yuan et al. 2007; Zhao 1992, 2007). In general, since S usually reduces the diversity of phytoplankton, the number of phytoplankton species in water with high S is less than in freshwater systems (Wang et al. 2023). Only the species with salt tolerance and adaptability in Bacillariophyta, Chlorophyta, and Cyanophyta can adapt to saline conditions (Chen et al. 2013). High S inhibits the growth of phytoplankton by altering their metabolic processes (Orizar et al. 2024). There is a significant negative correlation between the number of phytoplankton species and S. The ecosystems with high S are often dominated by a few species highly adapted to saline conditions, reducing the diversity index of the phytoplankton community. Nevertheless, the biomass of phytoplankton may be substantial since some salt‐tolerant species can witness explosive growth fueled by the nutrient supply, the species composition, and the environmental conditions (Orizar et al. 2024). S determines species composition and results in communities of low complexity, where few tolerant species ensure high biomass production in the absence of antagonistic interactions. This agrees with the findings in other studies (Afonina et al. 2025; Li, Gao, et al. 2021).

There was significant spatiotemporal variation in the structure of the phytoplankton community in Ebinur Lake. In terms of the temporal distribution, the biomass of phytoplankton in Ebinur Lake displayed considerable seasonal variation. In spring, the biomass of Bacillariophyta was dominant; in summer, as the temperature increased, the biomass of phytoplankton reached its peak, and the Chlorophyta was dominant; in autumn, the biomass was the lowest, and both Chlorophyta and Bacillariophyta shared dominance. In addition, the spatial distribution of phytoplankton remained relatively constant in the three seasons, with the southeast part of the lake generally exhibiting higher biomass levels than other lake areas. As there are salt flats in the southeast corner of the lake in addition to nearby farmland and salt fields, the spatial distribution characteristics of the lake may be associated with non‐point source pollution. During summer, sampling sites S18, S12, and S1 exhibited exceptionally high phytoplankton biomass, and this was correlated with their chlorophyll levels; the pattern was in large part due to the localized proliferation of the salt‐tolerant species *Dunaliella salina* at these sampling sites. The sampling points located at the shores of the lake are more susceptible to external environmental influence, enabling *Dunaliella salina* to rapidly proliferate under increased WT and abundant nutrient availability, thereby contributing to the generation of localized algal blooms (Wang et al. 2023).

### 4.2 Coupling Effects of Environmental Factors on the Structure of the Phytoplankton Community

Some studies showed that the environment is crucial in the formation of planktonic communities (Afonina et al. 2025; Golubkov et al. 2018; Zadereev et al. 2022). Ebinur Lake is characterized as a slightly alkaline saltwater body. The lake maintains an average S in the range of 10.05 to 14.55 and a pH between 8.08 and 8.22. The S level significantly influences the composition of phytoplankton species, as shown by the dominance of Bacillariophyta, Chlorophyta, and Cyanophyta. However, the S level exhibited a limited correlation with the fluctuations in phytoplankton biomass. The findings from the RDA and Mantel analyses further demonstrated that there was no significant correlation that exists between S and the biomass of various phytoplankton divisions and the biomasses of various species. This underscores the fact that although being a key factor affecting the community structure of phytoplankton, S is not the only limiting factor. Rather, the phytoplankton community structure is influenced by the interactions of S with other abiotic environmental factors and the competition and predation interactions among various trophic groups. Owing to the weak alkalinity of the water in Ebinur Lake, pH is a key environmental factor that affects the structure of the phytoplankton community, and the same conclusions can be drawn from the saltwater lake in the Onon‐Torey plain in Mongolia and five types of different lake in China (Afonina and Tashlykova 2018; Jia et al. 2022). The results revealed a considerable correlation between Cryptophyta in phytoplankton and pH. The summer pH (average = 8.08) was lower compared to spring (8.22) and autumn (8.13), and research has shown that the optimal pH for Cryptophyta ranges from 6 to 8. Therefore, the fact that the higher biomass observed in summer (0.0093 mg/L) compared to spring (0.0002 mg/L) and autumn (0.0029 mg/L) can be explained by pH. The increased pH can act as a potent negative factor for phytoplankton growth in cold alpine lakes (Jia et al. 2022). During summer, there was a significant negative correlation (*R* = −0.47, *p* < 0.05) between pH and NH_4_^+^‐N.

The RDA analysis identified a substantial positive correlation between *Dunaliella salina* and Chl‐a. The summer season saw increased Chl‐a compared to spring and autumn, with *Dunaliella salina* dominant in summer. In summer, Chlorophyta had the highest biomass, since *Dunaliella salina* belongs to Chlorophyta, where the content of Chl‐a was higher than that of other phyla of phytoplankton. The proliferation of *Dunaliella salina* in summer was correlated with increased S and WT. As a salt‐tolerant species, *Dunaliella salina* responds positively to higher S; as a member of the Chlorophyta, it has a significant positive correlation with the WT. The optimal growth temperature for Bacillariophyta is 10°C–25°C in temperate waters (Cohn et al. 2003), and Chlorophyta and Cyanophyta tend to have advantages at relatively higher temperatures (30°C–40°C) (Barati et al. 2019; Vu et al. 2020). There was a significant negative correlation between D and Chl‐a (*R* = −0.38, *p* < 0.05). The primary sources of Chl‐a in water bodies are phytoplankton and debris from aquatic vascular plants, with phytoplankton being the major contributor. Phytoplankton are affected by photosynthesis, and the upper layer of phytoplankton in the water is more suitable for growth, meaning that the phytoplankton are forced to move or slowly sink with the water flow and other hydrodynamic conditions.

The summer season witnessed a significantly lower SD in Ebinur Lake than in spring and autumn at only 0.16 m. Bacillariophyta and Chlorophyta were notably correlated with SD, and the RDA showed similar ecological adaptations of *Nitzschia stagnorum* , *Chroomonas* sp., *Achnanthes linearis* , and *Navicula viridula* , suggesting that they were more suitable for a growth environment with high transparency. Among the four algae, *Nitzschia stagnorum* , *Achnanthes linearis* , and *Navicula viridula* belong to Bacillariophyta. Some studies have suggested that the correlations between Bacillariophyta and the density distribution, flow velocity, and water transparency are significantly higher than those between nutrient levels and temperature (Lin et al. 2003). Studies have shown that compared to Cyanophyta and other phytoplankton, Bacillariophyta have a higher light requirement. When the water transparency decreases, light penetration into deeper waters is affected, and therefore, the growth of Bacillariophyta is reduced in the middle and lower layers (Orizar et al. 2024). This explains the relatively lesser dominance of Bacillariophyta in summer than in spring and autumn. In contrast, the Chlorophyta blooms in summer contribute to the reduced transparency of Ebinur Lake, reinforcing the observed significant correlation between Chlorophyta and SD.

Regarding nutrients, NH_4_^+^‐N also affects the structure of the phytoplankton community. Being an essential nitrogen source for photosynthesis and protein synthesis in phytoplankton, NH_4_^+^‐N is directly utilized by many phytoplankton species for growth. In contrast to NO_3_^−^, NH_4_^+^‐N is more readily absorbed and utilized by phytoplankton. In Ebinur Lake, NO_3_^−^ was relatively high, with an annual average of 2.19 mg/L. In aquatic environments with high S, the nitrification process is inhibited in some species (Chen et al. 2024; Li, Wang, et al. 2021). Therefore, NO_3_^−^ may not be readily consumed, leading to its accumulation in the lake and a potentially higher concentration compared to freshwater lakes. In general, a highly saline environment impedes the growth of many freshwater phytoplankton and microorganisms (Mo et al. 2021; Stankovic et al. 2024), and this may decelerate the absorption and transformation processes of NO_3_–N and thereby affect its concentration. Research on Qinghai‐Tibet Plateau lakes by Li, Gao, et al. (2021) also revealed that in saltwater lakes, elevated TN significantly inhibited the growth of dominant phytoplankton species, thereby narrowing the gap between dominant and subordinate taxa. This finding helps explain the seasonal dynamics in Ebinur Lake: TN concentrations are higher in autumn (7.64 mg/L), and this correlates with a decline in the dominance of *Dunaliella salina* during autumn. Consequently, the phytoplankton community structure shifts toward co‐dominance by Chlorophyta and Bacillariophyta.

Based on our results, we can infer the mechanisms by which physicochemical factors drive the seasonal variation in dominant phytoplankton taxa in Ebinur Lake. Salinity determines species composition and contributes to the simplicity of the community structure. Salinity and water temperature, in combination, promote the summer bloom of the dominant species *Dunaliella salina* , which accounts for elevated Chl‐a concentrations during this period. This bloom also reduces SD in summer, thereby decreasing the competitive advantage of diatoms, which prefer high light penetration. Higher summer pH likely favors the growth of Cryptophyta, explaining their increased biomass during this season. Elevated TN in autumn suppresses the growth of *Dunaliella salina* , reducing the dominance of Chlorophyta and narrowing the biomass gap between Chlorophyta and other phyla.

## 5 Conclusion

Compared to freshwater lakes, Ebinur Lake hosts a relatively smaller assemblage of phytoplankton species, while demonstrating a higher species diversity than other saline lakes. Bacillariophyta was the most prevalent phylum, followed by Chlorophyta and Cyanophyta. The phytoplankton community structure in Ebinur Lake undergoes significant spatiotemporal variation that is tied to seasonal variation and external environmental conditions. The coupling effects of various environmental factors, including pH, SD, Chl‐a, NH_4_^+^‐N, and S, affect the dynamics of the phytoplankton community structure in Ebinur Lake. This study examined the community structure of phytoplankton in Ebinur Lake, a representative saltwater lake in Xinjiang, China. The results revealed the ecological adaptations and species‐specific response mechanisms of phytoplankton under saline stress, as well as the coupled effects of environmental factors on phytoplankton community dynamics in saltwater lakes. These findings provide a scientific basis for water quality assessment, climate change response, and the management of lake biological resources. Moreover, they offer theoretical guidance for ecological interventions such as ecological water supplementation and saline‐alkali land restoration. The high‐salinity characteristics of Ebinur Lake also make it a valuable case study for research on plankton in extreme environments.

In future research, the authors will aim to gain a deeper understanding of the competition among different phytoplankton divisions, the predation pressure exerted by fish and zooplankton that feed on the phytoplankton, and the combined effects of biotic and abiotic environmental factors among these biological groups on the phytoplankton community structure. Additionally, the relationship between physiological and ecological characteristics of phytoplankton dominant species and environmental factors is also a research direction for the future.

Given climate change and human activities, Ebinur Lake is confronted with issues such as rapid lake shrinkage, water scarcity, and intensified salinization (Yang et al. 2024). If this situation worsens, the species of phytoplankton in Ebinur Lake, particularly those ubiquitous species with weak tolerance, will experience a gradual decline, thereby leading to decreased phytoplankton biodiversity. Accordingly, it is imperative to protect the ecological environment of Ebinur Lake by controlling the rational use of water resources, preventing the desertification of vegetation around the lake, and reducing non‐point source pollution, thereby ultimately safeguarding the biodiversity of phytoplankton and other aquatic organisms.

Accordingly, the following recommendations are proposed for the ecological protection and management of Ebinur Lake: establish a monitoring system for the lake's ecological environment and biological resources to provide a scientific basis for assessing the current state and trends of ecological change; implement ecological restoration projects such as re‐vegetation and reforestation (e.g., converting desertified or cultivated land back to grassland or forest) to enhance vegetation cover in the Ebinur Lake watershed; restore the lake's natural ecosystem primarily through natural regeneration, supplemented by targeted artificial interventions where necessary; develop and utilize biological resources sustainably, including scientifically planning the fishing seasons and zones for *Artemia salina* harvesting to ensure the long‐term viability of biotic resource use in Ebinur Lake.

## Author Contributions

**Huibo Wang:** data curation (equal), formal analysis (equal), investigation (equal), writing – original draft (lead). **Le Wang:** formal analysis (equal), investigation (equal), project administration (equal). **Xue Du:** data curation (equal), investigation (equal). **Xiaoli Huang:** formal analysis (equal). **Chen Zhao:** investigation (equal). **Zhongsi Gao:** formal analysis (equal). **Tangbin Huo:** data curation (equal), project administration (lead), writing – review and editing (lead).

## Conflicts of Interest

The authors declare no conflicts of interest.

## Supporting information


Data S1.

## Data Availability

All code and datasets used in the analysis are available at the following online repository (ScienceDB) (https://www.scidb.cn/en/s/NFBB7b).
